# DC Signature of Snap-through Bistability in Carbon Nanotube Mechanical Resonators

**DOI:** 10.1021/acs.nanolett.2c01187

**Published:** 2022-09-07

**Authors:** Sharon Rechnitz, Tal Tabachnik, Shlomo Shlafman, Michael Shlafman, Yuval E. Yaish

**Affiliations:** Andrew and Erna Viterbi Faculty of Electrical and Computer Engineering, Technion, Haifa 3200003, Israel

**Keywords:** MEMS, NEMS, carbon nanotubes, resonators, snap-through buckling, bistability

## Abstract

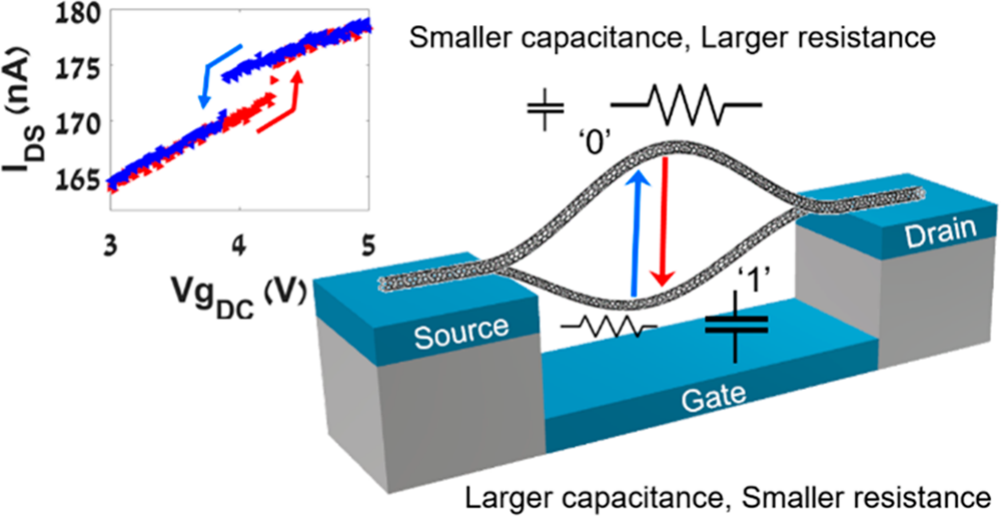

Bistable arched beams exhibiting Euler-Bernoulli snap-through buckling are widely investigated as promising candidates for various potential applications, such as memory devices, energy harvesters, sensors, and actuators. Recently, we reported the realization of a buckled suspended carbon nanotube (CNT) based bistable resonator, which exhibits a unique three-dimensional snap-through transition and an extremely large change in frequency as a result. In this article, we address a unique characteristic of these devices in which a significant change in the DC conductance is also observed at the mechanical snap-through transition. Through the analysis of this phenomenon, we arrive at several important conclusions: we find that the common approach to determining CNT vibrational resonance amplitude is inaccurate; we find evidence that latching phenomena should be easily realizable, relevant for RF switches and nonvolatile memory devices. Finally, we present evidence for possible inner shell sliding, which is relevant for understanding interlayer coupling and moiré pattern research.

An important feature for a micro/nanoelectromechanical system (MEMS/NEMS) is the ability to tune the state of the moveable object by an external parameter, and the most widely used is by electric fields. A common prototype is based on a conductive beam electrostatically actuated by applying a voltage difference between a nearby electrode and the movable beam.^1^ The applied voltage can alter the static position of the beam as well as excite its resonance modes.^2^ Numerous studies have examined the properties of such suspended beams under static and dynamic forces, including their linear and nonlinear behavior due to the combination of mechanical restoring force and electric field.^1,3−6^ Specifically, an arch-shaped beam which undergoes Euler-Bernoulli buckling instability is commonly used as a bistable device.^7−9^ In such an initially curved beam clamped at both ends and actuated by electrostatic force, a nonmonotonous stiffness-deflection characteristic is found, and snap-through (ST) buckling phenomenon can be observed.^10^ Under these circumstances, the mechanical constraint limits the beam movement, making the system stiffer after the ST transition, and an additional stable equilibrium appears. Such structures are suitable for various applications including sensors,^11^ memory devices,^12,13^ actuators,^14,15^ filters,^16^ microvalves,^17^ and buckling-induced smart applications.^18^

Recently, we reported the first realization of ST bistability in CNT resonators which results from initial upward buckling of the CNT.^19^ In this study, we present extensive data in which a discontinuity (“jump”) in the DC conductance measurement of the device is observed which is a signature of mechanical bistability. We analyze and explain the origin of this phenomenon, which turns out to be more complex than initially anticipated.

## Results

**Figure 1:**
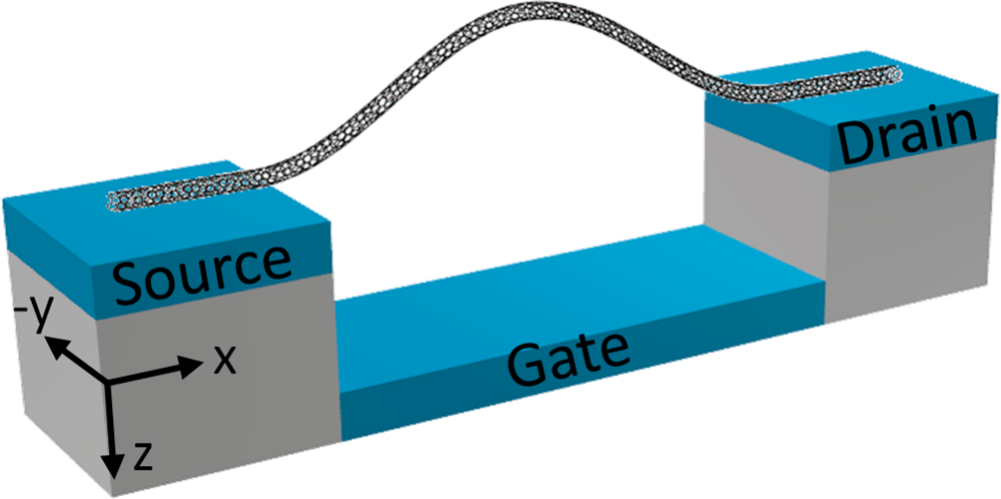
Schematic of a typical bistable CNT device with initial upward buckling and our coordinates system.

**Figure 2:**
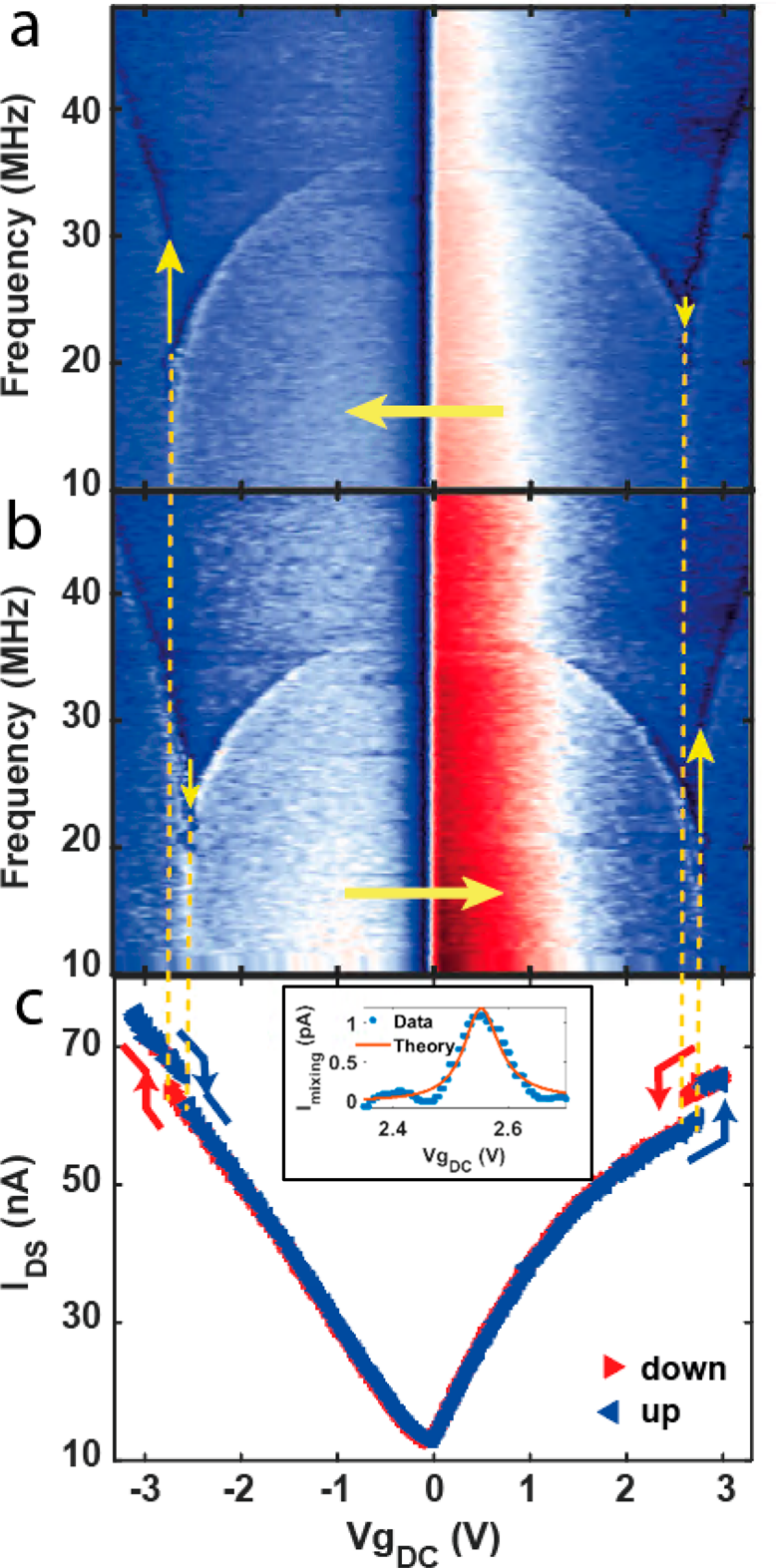
Conductance and resonance measurements of a typical bistable device. (a,b) Resonance frequency measurement of a typical device exhibiting snap-through bistability, consisting of downward (a) and upward (b) gate sweeps, marked by the horizontal yellow arrows. The abrupt transition from upward to downward curvature (and vice versa) is characterized by a jump in the resonance frequency, marked by the vertical yellow arrows. (c) Transfer characteristic curve of the same device as in (a,b), exhibiting standard small band gap carbon nanotube characteristics. The ST signature appears as a jump in the DC conductance at the same loads as the jumps and hysteresis in (a,b). Inset is a cross-section of B near the resonance current peak at *f* = 26 MHz with fit to a Lorentzian shape, from which the current peak is extracted.

**Figure 3:**
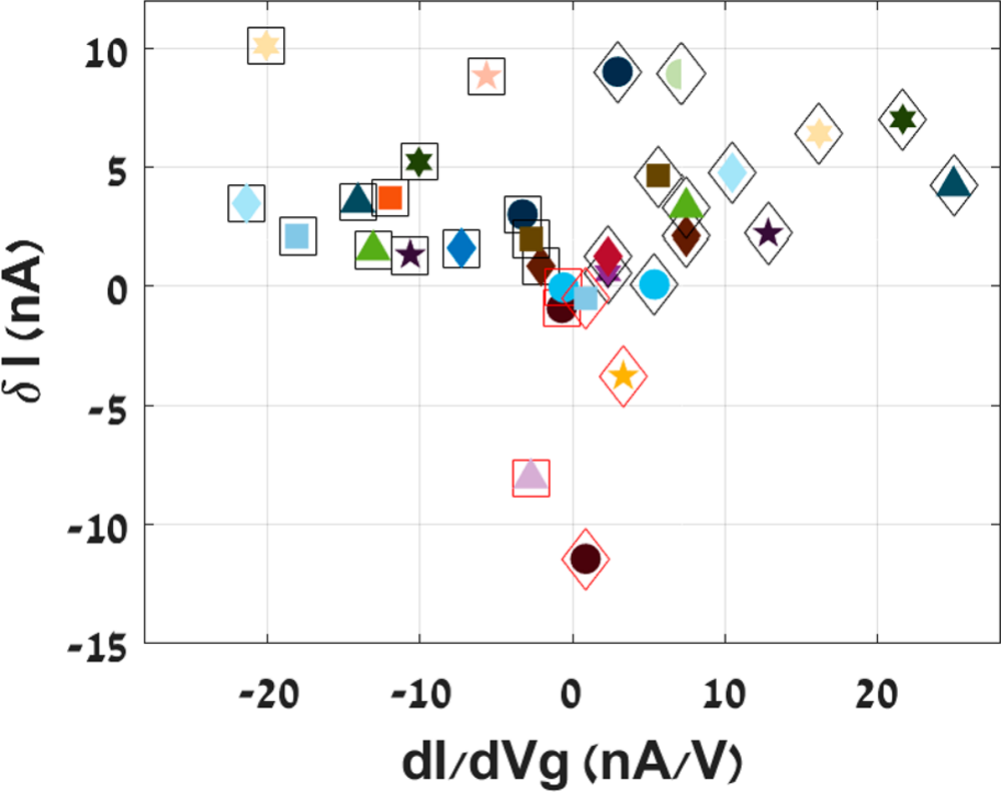
δ*I*_DS_ of jump vs ∂*I*/∂*V*_G_, acquired from 20 different bistable CNT resonators. Each device is characterized by marker type and color. The outer frame differentiates between jumps for p-type (square) or n-type (diamond) CNTs, and red frames indicate negative jumps in which a decrease in the conductance was observed.

Figure 1 presents a schematic of a typical bistable CNT resonator device discussed in this study. The bistability results from significant initial upward buckling and initial axial strain, induced by the fabrication process.^19^Figure 2a,b presents a resonance frequency measurement of a typical bistable CNT resonator for downward and upward DC gate sweep, respectively. The CNT is initially curved upward.^19^ As the gate voltage (absolute value) increases, the CNT is attracted toward the local gate, and the resonance frequency decreases due to compression. At a certain point (*V*_gDC,ST_ = 2.77 V for upward sweep or *V*_gDC,ST_ = −2.63 V for downward sweep), the CNT cannot compress any further and jumps to a downward configuration, a transition known as snap-through buckling. This mechanical transition results in a jump in the resonance frequency, marked by the vertical yellow arrows in Figure 2a,b. After the transition, increasing the gate voltages further stretches the CNT, and the resonance frequency increases. When the voltage is swept back to zero, the resonance frequency decreases as the stretching is gradually reduced, until reaching a second minimum, at which a snap-back (or “release”) transition occurs, also marked by the vertical yellow arrows (*V*_gDC,R_ = −2.47 V for upward sweep or *V*_gDC,R_ = 2.62 V for downward sweep). The difference between the gate voltages at which the ST (downward jump) and release (upward jump) transitions occur creates a hysteresis window. As an example for positive gate voltages, Δ*V*_hyst_ = *V*_gDC,ST_ – *V*_gDC,R_ = 0.15 V. We shall clarify that the actual CNT static motion is more complex than described in this simplified explanation, since an out-of-plane deflection also evolves.^19^ We developed a theoretical model that allows us to predict the exact CNT shape at every static load,^19,24^ but ultimately the snap-through jump represents a sudden transition from upward to downward configuration. Surprisingly, we observe a discontinuity in the DC transfer characteristic curves of bistable CNT resonators at the same load as the snap-through transition occurs (Figure 2c). Mechanical vibrations of a suspended CNT will usually have no effect on its DC conductance. However, since the Euler-Bernoulli snap-through transition results in a relatively large mechanical motion, it is also evident in a DC conductance measurement. Figure 3 presents data of similar DC jumps obtained from 20 different bistable CNT resonators. Each shape and color represent a device, where a square/diamond frame represents whether the jump occurs for p/n-type CNT, respectively. CNTs are classified as p-type or n-type, based on their majority charge carriers. For gate voltages smaller/larger than the Dirac voltage (at which the conductance is minimal), positive/negative charge carriers (holes/electrons) are induced and therefore the CNT is referred to as p-type/n-type. δ*I* is the DC current difference measured before and after the jump, plotted as a function of the DC measurement slope ∂*I*/∂*V*_G_. It can be observed that the majority of the jumps are positive (i.e., an increase in the conductance due to the ST transition), but occasionally we detect a negative jump (a decrease in the conductance). Note that negative jumps occur only for small ∂*I*/∂*V*_G_ values, meaning at saturation where there is nearly no conductance modulation due to change in the gate voltage, implying that the device conductance is mainly restricted by contact resistance.

In the following discussion we attempt to explain the ST DC jumps. The discussion will follow a single example, based on the device presented in Figure 2 (Device I), but we also present the results obtained in a similar manner to two other devices in the Supporting Information, arriving at the same conclusions.

## Discussion

### Naive Capacitance Model

Intuitively, we attribute the change in conductance to the change in capacitance due to the large mechanical snap-through transition. As a result, the charge induced upon the CNT by the local gate will change and hence the current
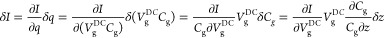
1where *q* is the charge induced upon the CNT, *C*_g_ is the capacitance between the CNT and the local gate, and the derivative is taken at constant gate voltage. *z* represents the in-plane CNT deflection, and δ*z* = |*z*_beforeST_ – *z*_afterST_| is the change of the CNT displacement due to the ST transition. The physical parameters of the device are detailed in Table 1.

**Table 1:** Physical Parameters of the Device Presented in Figure 2 (Device I).

symbol	physical parameter	AFM data
**g**_**0**_	height of the source and drain above the local gate	150 nm
**r**	CNT radius	1.3 nm
**L**	CNT length	1.6 μm

Taking the capacitance of a wire parallel to the plane and assuming  ≪ 1 (where *z* is the CNT deflection along the *z*-axis and *g*_0_ is the height of the source and drain above the local gate), we receive^24^. Assuming z≪g_0_, we obtain the relation
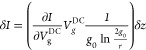
2

Hence, we should be able to estimate the mechanical jump δ*z* from the jump in the conductance. However, if we substitute all the parameters extracted from Figure 2c into eq 2, we get an estimation of *δz* ≈ 151 nm ≈ *g*_0_, which is inconsistent with our assumption.

**Figure 4:**
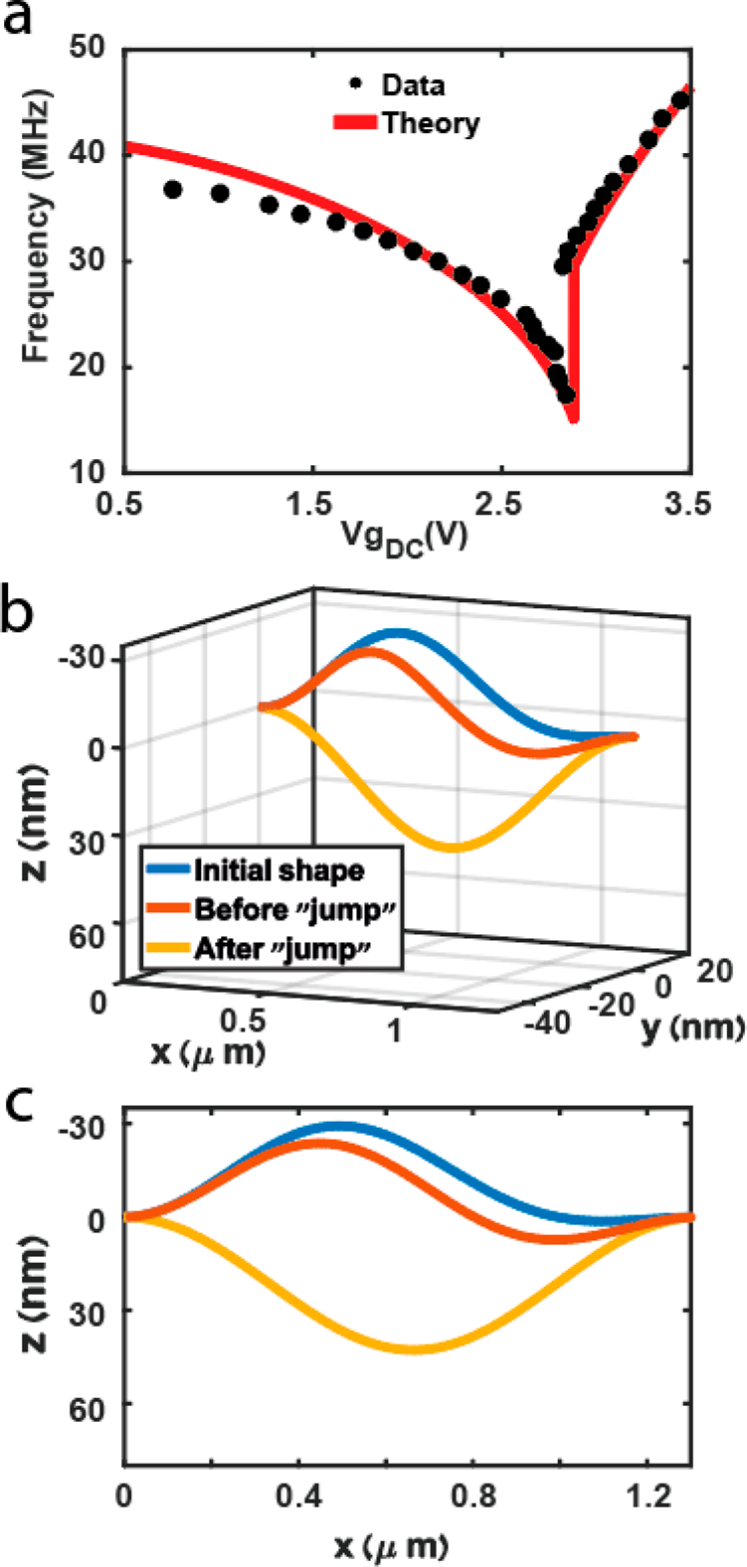
(a) Theoretical fit to the resonance data from Figure 2, according to the theoretical model in ref (19). (b) Three dimensional CNT shape at its initial configuration (blue) when no force is applied and just before (orange) and immediately after (yellow) the ST buckling transition. (c) In-plane component of the CNT configurations presented in (b), which is the only component affecting its capacitance to the local gate.

Fortunately, our theoretical model^19,24^ for the resonance frequency modes versus the gate voltage (Figure 4a) allows us to determine the exact shape and location of the CNT for any static load, and specifically before and after the ST transition (Figure 4b). Please note that our convention is such that the positive *z*-axis points downward. For capacitance calculations, only the in-plane (*z*) component is relevant (Figure 4c). Examining the in-plane CNT shape raises the question of how δ*z* should even be estimated. Before we answer this question, we shall notice that even if we consider the maximum deflection *δz*_max_ = max(*z*_down_(*x*)) – min(*z*_up_(*x*)) = 52.9 nm, it can only account for a jump of *δI* = 1.45 nA, which is only a quarter of the experimental *δI*_exp_ = 4.77 nA. Therefore, taking a more realistic δ*z* of, for example, the average displacement (i.e., *δz* = avg(*z*_down_(*x*)) – *avg*(*z*_up_(*x*))) will also fall short. In addition, the capacitance model cannot account for the negative jumps (a decrease in the conductance as a result of the ST transition) occasionally detected (Figure 3 and Figure S1).

### Strain-Induced Conductance Modulation

The mechanical ST transition involves also a sudden change in the axial tension along the tube, evidenced also as the jump in frequency (Figure 2a,b). Strain can both enlarge as well as reduce the band gap,^20−22^ which can potentially explain both an increase as well as a decrease in the conductance, as observed in the data. We explore this theory in detail in Supporting Information and find that the change in tension due to the snap-through transition can only predict a jump in current which is 2 orders of magnitude smaller than the jump measured in the experiment.

Taking a closer look at Figure 3, we notice that the negative jumps occur only when d*I*/d*V*_g_ is small, i.e., when the contact resistance is more dominant than the intrinsic CNT resistance (see Figure S1 as an example). This indicates that the jump might in fact be related to the contacts. We investigate this option in detail in the Supporting Information as well and conclude that the effect of Schottky barrier modification due to the change in strain can also only predict a jump in current which is 2 orders of magnitude smaller than the jump measured in the experiment. Even if we consider both effects that arise from the band gap modification as a result of strain, they still cannot account for the experimental jump in current.

### Modified Capacitance Analysis

Since tension cannot account for the jumps measured in the experiments, we turn back to the more intuitive explanation, that the jump in the current results from the change in capacitance due to the mechanical ST. The above model (eq 1) assumes . However, we know that the capacitance is not constant and changes with the CNT movement. We wish to estimate the change in current due to the partial derivative of the current with respect to the capacitance, i.e., to utilize the following relation
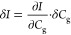
3

**Figure 5:**
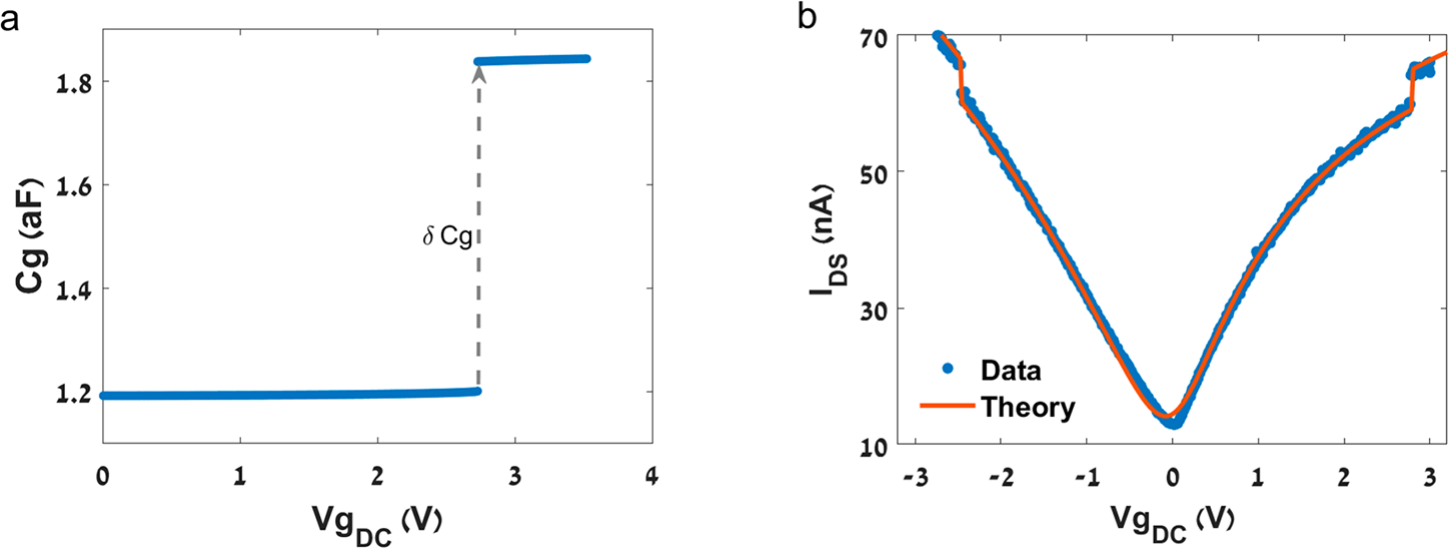
Modified capacitance analysis. (a) Gate capacitance as a function of the gate voltage, calculated according to eq 4, where *z*(*x*) was obtained from the theoretical fit in Figure 4a for every static load. (b) Theoretical fit according to eqs S1–S6, where *C*_g_(*V*_g_) was extracted from (a). The theoretical jump estimations according to the modified capacitance model were added manually at the ST gate voltages.

First, since we know the exact CNT shape (*z*(*x*)) for every static load, we can calculate the capacitance between the CNT and the local gate for every load according to
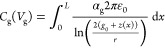
4where *ε*_0_ is the vacuum permittivity.  is the gate efficiency factor,^23^ and in order to be consistent we use the same value received from the resonance frequency fit, which is mostly determined by the gate voltage at which the ST transition occurs. The calculated capacitance gate dependence according to eq 4 is depicted in Figure 5a, from which we extract the capacitance difference due to the ST buckling transition: *δC*_g_ = 0.64 aF.

Next, in order to estimate the partial derivative , we return to our conductance model (eqs S1–S6), and substitute the real *C*_g_(*V*_g_) to correct our theoretical fit to the data (Figure 5b). Then, we add a 3% change in the capacitance, solve the equations once more, and estimate the partial derivative as the difference between the new current and the original current at the same DC voltage at which the jump occurs (in our example, *V*_g*,*ST_ = 2.77 *V*): .

Substituting into eq 3 yields an anticipated *δI*_V_g,ST_=2.77V_ = 5.8 ± 0.1 nA, which is in good agreement with the measured jump in the current, *δI*_exp,1_ = 4.77 nA. We do the same for the jump at *V*_g*,*ST_ = – 2.42 V and obtain *δI*_V_g,ST_=–2.45V_ = 6.2 ± 0.1 nA, also in relatively good agreement with the experimental *δI*_exp,2_ = 4.35 nA (Figure 5b).

### Capacitance Calculation

In order to verify our capacitance estimation, we also model our device geometry in COMSOL (Figure S3). A full electrostatic analysis with the calculated CNT configuration (*z*(*x*)) after the transition yields *C*_g,down_^COMSOL^ = 1.083 aF, fairly close to our estimated *C*_g,down_^Eq.12^ = 1.2 aF. For the upward configuration we obtain: *C*_g,down_^COMSOL^ = 1.765 aF, in good agreement to our estimated *C*_g,down_^Eq*.*12^ = 1.84 aF. These results are extremely encouraging. First, they affirm that our estimation of α_g_ from the resonance fit is reasonable for this device geometry. Second, if we use *C*_g_^COMSOL^ = 0.682 aF, we get a prediction of *δI*_V_g,ST_= 2.77V_ = 6.26 nA, which is still comparable to the experimental jump and consistent with our estimation.

### Implications of the Modified Capacitance Model

We would like to emphasize the significance of the fact that the modified capacitance must be considered to obtain a good prediction. Whenever using the mixing technique to measure the resonance frequencies of a CNT resonator,^25^ the naive approach has been the popular way of estimating the vibrational amplitude at resonance, according to , where *δI*_mix_ is the mixing current peak at the resonance frequency.^25,26^ Vibrational amplitude is an extremely important characteristic of a resonating device, and it is crucial for vibrational analysis.^27^ In this study, we show that estimating  as was customary is incorrect, since the capacitance contribution to the derivative is not at all negligible.

The accurate calculation should be
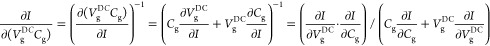
5

Hence, the estimation that  is correct only under the assumption that . When comparing these two terms in our device before the ST transition, when the motion is continuous, as can be seen in Figure S4, the two terms in the denominator are comparable, and the assumption is incorrect.

To prove our claim, let us examine the effect of this correction on vibrational analysis. We wish to estimate the vibrational amplitude of the CNT at resonance. From a Lorentzian shape fit to the mixing current peak at *f* = 26 MHz (inset of Figure 2c), we extract *I*_peak_ = 1.0089 pA. Using the traditional, naive, method, we extract 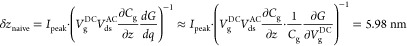
However, if we calculate  according to eq 5, we obtain 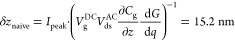
This means that whenever the mixing current was used to estimate the vibrational amplitude of CNT resonators, it was inaccurate, and therefore so are the results obtained using this method in estimation of damping, nonlinear coefficients, symmetry breaking, and so forth.^25−30^ Analysis of two-dimensional material-based resonators is also commonly based on the mixing technique and hence should probably also be reevaluated according to our conclusions.^31^

### Unsolved Mysteries

We present the results of a similar analysis from two additional bistable devices in the Supporting Information (Table S1) from which we can also deduce that the modified capacitance model achieves the best prediction. We believe that the change in capacitance is the dominating mechanism affecting the CNT conductance at the ST transition. We were able to achieve good predictions for several devices in which the jump occurs at gate voltages for which the CNT intrinsic resistance is most dominant (Table S1). Unfortunately, negative jumps remain a puzzle. As stated before, we observe that negative jumps occur only when the current is roughly constant with respect to the gate voltage, and the contact resistance is most dominant in setting the total CNT resistance. Therefore, we believe that there is another mechanism in which the ST transition also affects the contact (either competing or contributing). Further investigation of the data reveals that negative jumps were only observed in devices with relatively thick CNT diameters (*d* ∼ 4–6nm, measured in AFM). This implies that these CNTs are likely to have two or more walls. Hence, one possibility for a contact effect could be inner-shell sliding^32,33^ relative to the fixed outer shell which adheres to the metallic electrodes due to stretching. Such sliding is thought to modify the contact resistance and can either increase or decrease the CNT current. We are currently pursuing this direction, and further experiments are needed in order to explore this hypothesis. If inner-shell sliding occurs, this can be very appealing for the study of interaction between shells and moiré pattern changes that might occur as a result.

### Large Hysteresis Window

Most jumps are also characterized by noticeable hysteresis, as predicted by ST theory. There is no clear correlation between the jump height, δ*I*, and the hysteresis window size (Figure S5a), even though both parameters are essentially dependent on the initial CNT configuration. Nevertheless, it is understandable, as the jump height also depends on the CNT conductivity, which is independent of the initial configuration. In a few cases, we encounter a device with a very large relative hysteresis window, Δ*V*_G,hyst_/*V*_G,ST_ ∼ 1, as in the transfer characteristics in Figure S5b. Latching refers to a nonvolatile trait of a bistable buckled beam device in which the beam remains in the downward configuration after the electrostatic force is removed.^34^ Our theory predicts that under certain initial conditions, latching should be realizable in CNT bistable devices.^24^ The latching criterion is usually formalized by the demand that the force at which a snap-back occurs must be negative (*F*_SB_ < 0). However, if we define the relative hysteresis window as Δ*V*_G,hyst_/*V*_G,ST_, the latching criterion can also be written as Δ*V*_G,hyst_/*V*_G,ST_ > 1. In our devices, we cannot test this theory, as we only have a single local gate, and therefore cannot apply a repulsive force to observe the snap-back of the device. However, if we consider the hysteresis criterion, we know that it is definitely in reach, as we have observed relative hysteresis windows approaching 1 (orange dots in Figure S5a).

## Conclusions

In summary, we present experimental data, signature of bistable CNT resonators, in which mechanical ST transition is accompanied by a change in the DC conductance of the device. We attempt to explain the seemingly intuitive phenomenon by several models. We show that the change in strain cannot account for the significant DC jumps measured, neither because of tension modulation of the contacts resistance, nor because of modulation of the intrinsic CNT conductance. We conclude that the dominant mechanism causing the jump is the change in capacitance, which is not trivial to predict without knowing the exact position and shape of the CNT. Together with a Drude-based model for the CNT conductance, we calculate the contribution of the capacitance change alone to the current and obtain excellent agreement with the experimental data. The significance of the capacitance change for achieving a good prediction implies that the commonly used method to estimate vibrational amplitude from resonance measurements based on the mixing technique is inaccurate. We show that the capacitive model cannot account for all the experimental data, and specifically a decrease in the conductance at the ST transition. We attribute this decrease to a change in the contacts, possibly inner shell sliding, but this hypothesis has not been experimentally validated. We believe that the profound understanding of the conductance jumps of bistable CNT resonators can lead to better and more robust design of such devices, and we present the indication of realistically achievable latching devices as an example.

## Methods

### Device Fabrication

All CNT resonators were fabricated according to the local gate self-aligned technique reported in ref (35). Briefly, source and drain electrodes were patterned using standard e-beam lithography, and Cr/Pt 7/32 nm metal layers were evaporated. After the SD patterning, a critical BOE etching step is required,^19^ after which the local gate was patterned self-aligned, and another layer of Cr/Pt 7/32 nm metals were evaporated. Finally, CNTs were grown in CVD using the fast heating growth technique^36^ with H2/CH4 0.5/0.5 SLM flow at 900 °C.

### Conductance Measurements

All conductance measurements from which the data in Figure 3 was extracted were conducted in vacuum of *P* ∼ 4 × 10^–4^ Torr at room temperature. A *V*_DS_ = 10 mV bias was applied between the source and drain, and the current was measured using a Stanford SR-570 low noise current preamplifier.

## Supporting Information Available

Results summary from additional devices; analysis of the band gap modulation due to strain; analysis of the Schottky barrier modulation due to strain; Figure S1, negative DC jump; Figure S2, Schottky barrier modulation due to strain; Figure S3, device geometry modeling in COMSOL for capacitance estimation; Figure S4, conductance derivative with the charge; Figure S5, relative hysteresis window histogram (PDF)

## ABBREVIATIONS

DC: direct current
CNT: carbon nanotube
ST: snap-through
