# Supplementary material for: DC Signature of Snap-through Bistability in Carbon Nanotube Mechanical Resonators
Source: Nano Lett. 2022 Sep 7;22(18):7304–10. doi: 10.1021/acs.nanolett.2c01187 (PMC9523715; doi:10.1021/acs.nanolett.2c01187)
Supplement: Supplementary file 1 — nl2c01187_si_001.pdf [file nl2c01187_si_001.pdf]

DC Signature of snap-through bi-stability in carbon nanotube mechanical resonators

Supporting Information

AUTHORS: Sharon Rechnitz, Tal Tabachnik, Shlomo Shlafman, Michael Shlafman and Yuval E. Yaish.*

Andrew and Erna Viterbi Faculty of Electrical and Computer Engineering, Technion, Haifa, Israel.

Additional Devices

Following is a table summarizing the analysis in the main text discussion and additional two small band-gap devices exhibiting a positive DC ST “jump” that were analyzed in the same manner as described in the main text:

Device	δI_{exp} Experimental "jump"	Naïve capacitance model	Band gap modulation due to strain	Schottky barrier modulation due to strain	Modified capacitance analysis
I	4.77 nA	1.45 nA	52 pA	30.2 pA	5.8 nA
II	1.3 nA	0.88 nA	48 pA	29.3 pA	1.6 nA
III	3 nA	1.38 nA	80 pA	62.1 pA	3.7 nA

Table S1. Results summary of the predicted "jump" from the different theories discussed in the text compared to the experimental "jump" for three different CNT devices.

It can be easily observed that the conclusions presented in the main text for Device I are also valid for devices II and III, and can therefore be generalized.

Modulation of the band gap due to strain

Since we know the exact shape of the CNT for every static load, we can calculate the tension along the tube as well as the strain, $\epsilon = \frac{\Delta L}{L_0} = \frac{T}{EA}$, where T is the axial tension, E is the CNT Young's modulus, A is the cross section area, L_0 is the relaxed CNT length (if no tensile forces are applied), and ΔL is the change in the CNT length due to strain. For the CNT in Fig. 4 we extract $T_{up} = 0.1339\, nN$, the equivalent of $\epsilon_{up} = 2.8838 \cdot 10^{-5}$, and $T_{down} = 0.1276\, nN$, the equivalent of $\epsilon_{down} = 2.7476 \cdot 10^{-5}$, meaning that the change in strain as a result of the ST transition is $\delta\epsilon = 1.3623 \cdot 10^{-6}$. The band gap dependence on the strain[20] is given by $\delta E_g = \pm (3t_0(1+\nu)\cos(3\phi)) \cdot \delta\epsilon$, where t_0 is the tight-binding overlap integral, ν is the Poisson ratio, and ϕ is the CNT chiral angle. As an upper bound estimation, we substitute typical values from Refs. [21,22] ($t_0 \approx 2.7$ and $\nu \approx 0.2$) and assume armchair CNT ($\cos(3\phi)$=1). This results in a change in the band gap of $|\delta E_g| \approx 1.3242 \cdot 10^{-5} eV$. How should this translate into a change in the CNT conductance?

In order to estimate the resulting expected change in current (δI), we need to know the band gap of the device. For this purpose, we have developed a Drude-based theoretical model for the current gate dependence for single-wall CNT, as depicted in Fig. 2. We begin with the basic relation:

$$I = G_T V_{DS} = (G_T^n + G_T^p) V_{DS} \quad \text{(Eq. S1)}$$

where V_{DS} is the voltage difference between the two contacts, and G_T is the total conductance, which is the sum of the electrons' conductance and the holes' conductance, each comprised of the intrinsic CNT conductance in series with the contact resistance:

$$\left(G_T^{n/p}\right)^{-1} = \frac{L}{\sigma_{n/p}} + R_{Cn/p} \qquad \text{(Eq. S2)}$$

where $\sigma_{n/p}$ and $R_{Cn/p}$ are the CNT conductivity and the contacts resistance for electrons/holes, respectively. The CNT conductivity is estimated according to the Drude model:

$$\sigma_{n/p} = e \cdot \mu_{n/p} \cdot n(V_g)/p(V_g) \quad \text{(Eq. S3)}$$

where $\mu_{n/p}$ is the mobility and n/p is the number of negative/positive charges per length, which is a function of the gate voltage, given by:

$$n = \int_{\Delta}^{\infty} \nu(\varepsilon) f_{FD}(\varepsilon - \tilde{\mu}) d\varepsilon$$

$$p = \int_{-\infty}^{-\Delta} \nu(\varepsilon) f_{FD}(\tilde{\mu} - \varepsilon) d\varepsilon \quad \text{(Eq. S4)}$$

where f_{FD} is the Fermi-Dirac distribution, $\tilde{\mu}(V_g)$ is the chemical potential, ε is energy, Δ is half of the band gap ($\Delta = E_{gap}/2$), and ν is the density of states given by:

$$\nu(\varepsilon) = \frac{4}{\pi \hbar v_F} \frac{\varepsilon}{\sqrt{\varepsilon^2 - \Delta^2}}$$

where v_F is the Fermi velocity.

When non-zero voltage is applied to the gate, the CNT electric potential (φ_{NT}) is given by:

$$e\varphi_{NT} = \frac{e^2(n-p)}{C_T} - \frac{eC_g}{C_T} V_g \quad \text{(Eq. S5)}$$

where C_T,C_g are the total and gate capacitances per length, respectively, and $\alpha_g = \frac{C_g}{C_T}$ is the gate efficiency factor, which can be extracted from our fitting to the resonance measurement (more details in the "Modified capacitance analysis" section below). If the source-drain (SD) bias is negligible ($V_{DS} \ll \tilde{\mu}$), we can assume that both source and drain are at zero electro-chemical potential and therefore $e\varphi_{NT} + \tilde{\mu} = 0$. Substituting into Eq. S5, we obtain the relation:

$$V_g = \frac{e(n-p)}{C_g} + \frac{\tilde{\mu}1}{e\alpha} \qquad \text{(Eq. S6)}$$

We solve Eqs. S6 and S4 self-consistently and substitute into Eqs. S1-S3 to calculate $I(V_g)$. We find the physical parameters of the device from fitting the theoretical $I(V_g)$ to our conductance measurement (Fig. 5b), from which we obtain a band gap of E_g=192 $\pm$ 5 meV. Then, we substitute the modified band gap $\widetilde{E_g} = E_g \pm \delta E_g$, in which δE_g is our upper-bound estimation for the tension induced band gap modulation, into Eq. S4, solve the equations again self-consistently, and obtain how the current should change. Unfortunately, when substituting the above-estimated δE_g, we anticipate a change of only $|\delta I| = 52\ pA$. A back-of-the-envelope calculation gives $\delta I \approx I_0 \cdot \delta E_g / (2k_B T) \approx 100\ pA$, which agrees well with the more accurate calculation.

Modulation of the Schottky barrier as a result of strain

Since change in strain can enlarge or reduce the band gap, it can also increase or decrease the Schottky barrier (Fig. S2). Assuming thermionic emission, $R_c \propto e^{-\frac{\Phi_B}{k_B T}}$, where Φ_B is the barrier height, k_B is the Boltzmann constant, and T is the temperature. Since $\delta\Phi_B = \delta E_g \ll E_g$, we can approximate $e^{-\frac{\delta\Phi_B}{k_B T}} \approx 1 + \beta \cdot \delta\Phi_B$ ($\beta = \frac{1}{k_B T}$), and the resulting modulation of the contact resistance should change as $\delta R_c \propto e^{-\beta\Phi_B} \cdot \beta\delta\Phi_B$. Hence, we obtain the relation:

$$\frac{\delta R_c}{R_c} = \beta \cdot \delta E_g \qquad \text{(Eq. S7)}$$

Assuming saturation, $R_{total} \approx R_c$, the expected change in current is thus given by:

$$\delta I = -I \cdot \frac{\delta R_c}{R_c} \qquad \text{(Eq. S8)}$$

Using $|\delta E_g| \approx 1.3242 \cdot 10^{-5} eV$, as estimated above, we receive an anticipated "jump" of only $|\delta I| = I \cdot |\delta E_g| \cdot \beta \approx 30.42\ pA$.

Supplementary Figures

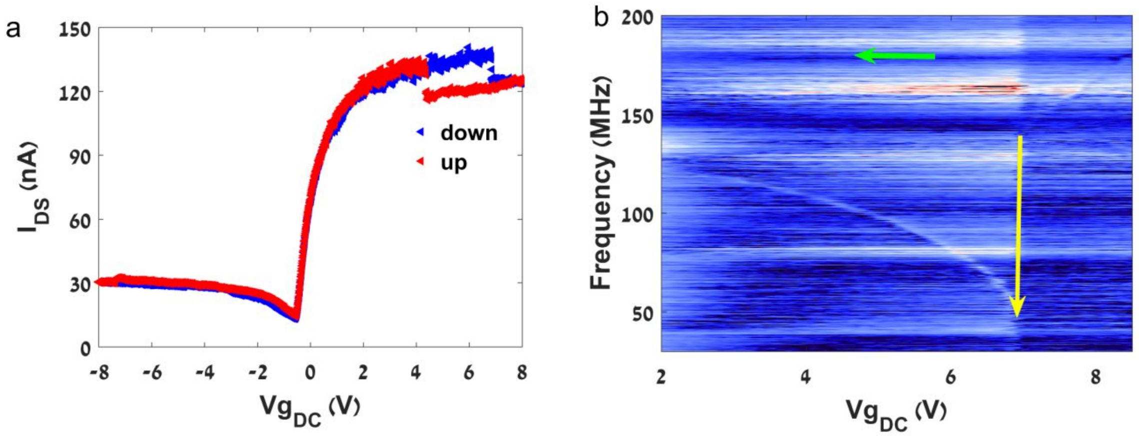

Figure S1. (a) Transfer characteristics curve exhibiting a decrease in conductance at the snap-through transition ("negative jump"). (b) Resonance frequency measurement (downward sweep,

as indicated by the green arrow) of the same device as in (a) confirming that ST transition (marked by the yellow arrow) occurs at the same gate voltage as the conductance "jump" in (a).

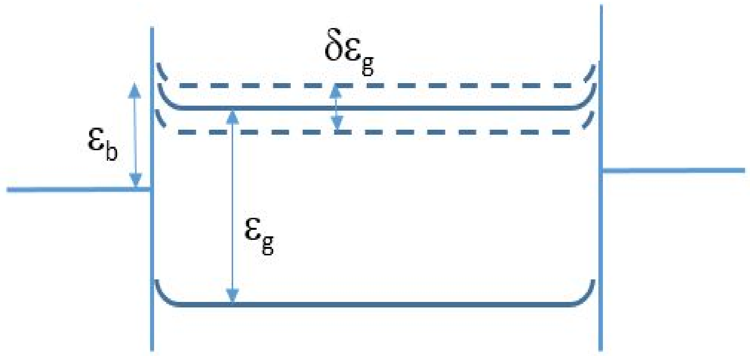

Figure S2. Schematic illustration of the Schottky barrier modulation as a result of the band gap modulation due to strain.

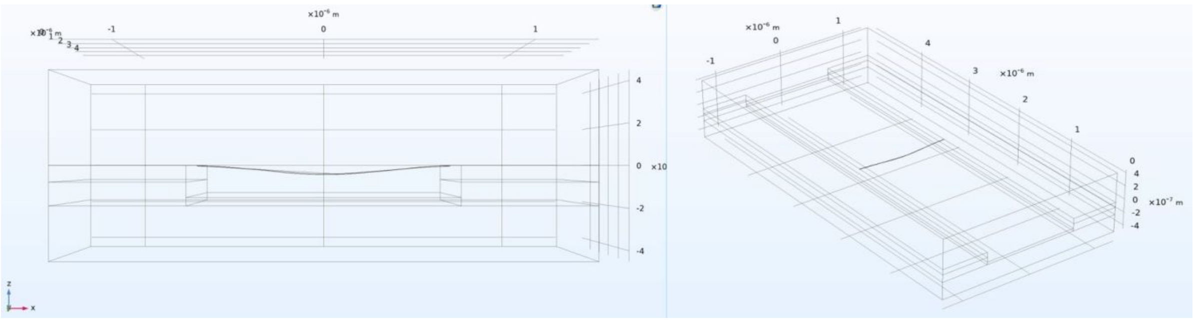

Figure S3. Device geometry modelling in COMSOL for capacitance estimation.

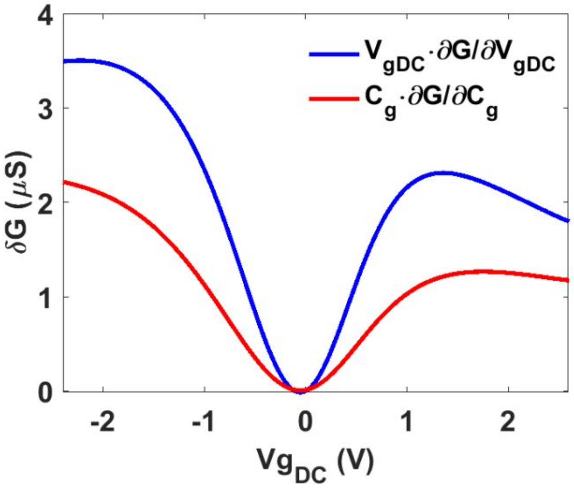

Figure S4. A comparison between the two terms in the denominator of the accurate expression for dG/dq (Eq. 5) as a function of V_{gDC}, showcasing that the assumption $V_g^{DC}\frac{\partial I}{\partial V_g^{DC}} \ll C_g\frac{\partial I}{\partial C_g}$ is incorrect.

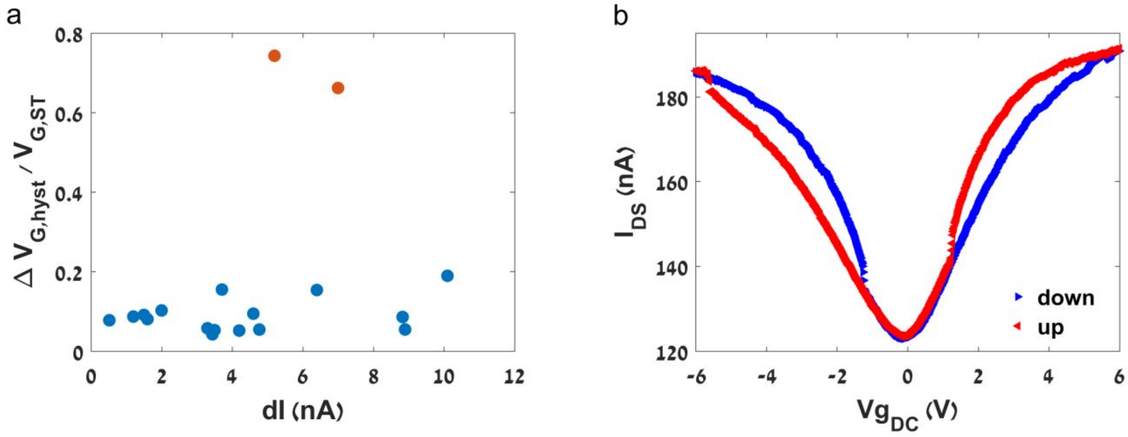

Figure S5. (a) Relative hysteresis window as a function of "jump" height. The orange dots represent a relative hysteresis window approaching 1. (b) Conductance measurement of a bi-stable device with a large hysteresis window approaching latching.
